# GmTRAB1, a Basic Leucine Zipper Transcription Factor, Positively Regulates Drought Tolerance in Soybean (*Glycine max*. L)

**DOI:** 10.3390/plants13213104

**Published:** 2024-11-04

**Authors:** Hui Li, Qiu-Yu Zhang, Ping Xu, Xiao-Hua Wang, Sheng-Jie Dai, Zhen-Ning Liu, Meng Xu, Xue Cao, Xiao-Yu Cui

**Affiliations:** College of Agriculture and Forestry Sciences, Linyi University, Linyi 276000, China; lihuiqau@163.com (H.L.); zhangqiuyu1025@163.com (Q.-Y.Z.); xuping3792@lyu.edu.cn (P.X.); wangxiaohua@lyu.edu.cn (X.-H.W.); daishengjie@lyu.edu.cn (S.-J.D.); liuzhenning@lyu.edu.cn (Z.-N.L.); xumeng@lyu.edu.cn (M.X.); caoxue@lyu.edu.cn (X.C.)

**Keywords:** soybean, drought, ABA response, ROS homeostasis, bZIP transcription factor

## Abstract

The basic leucine zipper (bZIP) transcription factors play crucial roles in plant resistance to environmental challenges, but the biological functions of soybean bZIP members are still unclear. In this study, a drought-related soybean bZIP gene, *GmTRAB*1, was analyzed. The transcript of *GmTRAB1* was upregulated under drought, ABA, and oxidative stresses. Overexpression of *GmTRAB1* improved the osmotic stress tolerance of transgenic *Arabidopsis* and soybean hairy roots associated with increased proline content and activity of antioxidant enzymes and reduced accumulations of malonaldehyde and reactive oxide species. However, RNA interference silencing of *GmTRAB1* in the soybean hairy roots improved drought sensitivity. Furthermore, GmTRAB1 increased the sensitivity of transgenic plants to ABA and participated in modulating ABA-regulated stomatal closure upon drought stress. In addition, GmTRAB1 stimulated the transcript accumulation of drought-, ABA-, and antioxidant-related genes to respond to drought. Collectively, this research will contribute to understanding the molecular mechanisms of bZIP transcription factors in soybean’s resistance to drought.

## 1. Introduction

Plants usually encounter multiple adverse environments, including extreme temperatures, drought, and salt, and these stresses can greatly affect their growth and development and ultimately destroy their productivity [1]. To survive, plants have evolved a variety of strategies to cope with these environmental challenges. These adaptive responses are highly intricate processes mediated by sophisticated signaling networks [2]. Transcription factors, including those from families including dehydration-responsive element-binding (DREB), myeloblastosis (MYB), NAM, ATAF, and CUC (NAC), WRKY, ethylene-responsive factor (ERF), and basic leucine zipper (bZIP), play critical roles in the signal transduction networks, and they function essentially in transmitting the perceived stress signal to the stress-responsive genes, ultimately leading to physiological and metabolic changes [3,4,5].

The bZIP transcription factors comprise a large and diverse transcription factor family in plants [6]. Members of bZIP proteins have a conserved bZIP domain, which consists of a leucine zipper motif and a conserved base region [7]. The N-X7-R/K motif is composed of approximately 18 amino acids within the bZIP domain and performs critical functions in DNA binding and nuclear localization [8]. The bZIPs usually interact with the promoter fragments harboring an ACGT core *cis*-element, including C-box (GACGTC), ABRE (CCACGTGG), A-box (TACGTA), and G-box (CACGTG), to influence the expression of downstream targets [9]. The leucine zipper region comprises one or more repeating regions or other hydrophobic amino acids, which is correlated to bZIP recognition and dimerization [10]. The bZIP proteins were first characterized in *Arabidopsis*, and 75 bZIPs were identified [7]. Subsequent genomics research also found 89 bZIPs in rice [10], 89 bZIPs in barley [11], 55 bZIPs in tomato [12], 247 bZIPs in rapeseed [13], 92 bZIPs in sorghum [14], and 125 bZIPs in maize [15].

The bZIP proteins perform crucial functions in plants’ responses to adverse conditions. For example, in *Arabidopsis*, AtbZIP1 increased the expression of *COR15A*, *RD17*, and *RD29A* to enhance *Arabidopsis’* ability to tolerate salt and osmotic stresses [16]. AtABF3 regulated ABRE-dependent gene expression to influence ABA-mediated drought response [17]. In rice, OsbZIP23, OsbZIP42, and OsbZIP72 were upregulated by ABA and drought stresses and act as key regulators in rendering rice ABA-regulated osmotic stress tolerance [18,19,20]. ZmbZIP4 has been reported to increase ABA synthesis to improve maize adaptation to drought and salt stresses [6]. Under cold conditions, ZmbZIP68 restricts the expression of DREB family proteins to reduce cold tolerance of maize [2]. TabZIP60 is associated with TaCDPK30 to influence the synthesis of ABA and increase the salt tolerance of wheat [5]. *TabZIP15* was shown to respond to salt stress, and *TabZIP15* overexpression conferred the resistance of transgenic wheat to salt [21]. Overexpression of *CsbZIP18* impaired the cold tolerance of transgenic plants through suppressing cold- and ABA-related genes’ expression [22]. Furthermore, in pepper, salt promoted *CabZIP25* expression, and *CabZIP25*-overexpressing (OE) plants displayed salt-tolerant phenotypes [23].

Soybean is a critical cereal and oil crop worldwide [24,25]. Drought acts as the main adverse stimulus to destroy the production of soybean [26]. Studying the mechanism in drought response is an effective way to enhance the adaptability of soybean to drought. Previous studies have reported that there are 160 bZIP genes in the soybean genome [27]. Until now, only a very limited number of bZIP members have been functionally analyzed in soybean. In the present research, we characterized a bZIP family transcription factor, GmTRAB1, and show that its expression is significantly upregulated upon drought, ABA, and oxidative stresses. Further physiology and molecular studies displayed that GmTRAB1 influences the ABA response and antioxidant defense system to enhance soybean tolerance to drought.

## 2. Results

### 2.1. Identification and Sequence Analysis of GmTRAB1

**Figure 1:**
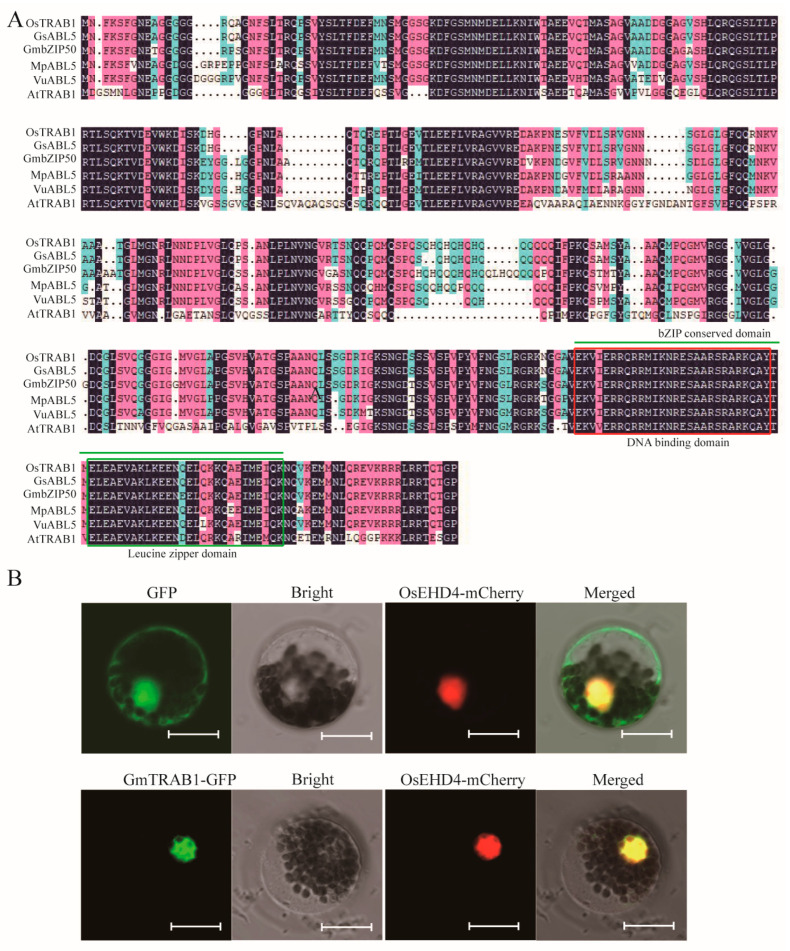
Sequence and localization assay of GmTRAB1. (**A**) Structure analysis of GmTRAB1, GsABL5, GmbZIP50, MpABL5, VuABL5, and AtTRAB1. The bZIP conserved domain is noted with dark lines. The DNA binding domain and leucine zipper domains are marked with red and green rectangles, respectively. (**B**) The subcelluar localization of GmTRAB1. OsEHD4-mCherry is referred to as a nuclear marker. Bar = 12 µm.

Results of bioinformatics analysis have shown that 160 bZIP transcription factors were identified in soybean [27]. GmTRAB1 (Glyma.04G039300) encoded a protein with a conserved bZIP domain and highly shared the sequence with GsABL5 from *Glycine soja* (99.52%), GmbZIP50 (89.35%), MpABL5 from *Mucuna pruriens* (87.97%), and VuABL5 from *Vigna unguiculata* (86.35%) (https://blast.ncbi.nlm.nih.gov/Blast.cgi?CMD=Get&RID=JEV01RW2016, accessed on 31 October 2024; Figure 1A). Subsequent phylogenetic analysis demonstrated that GmTRAB1 and its homologous proteins were classified into subgroup A (Figure S1). In the subcellular localization analyses, GmTRAB1-GFP proteins were detected in the nucleus and overlapped with the nuclear marker (OsEHD4-mCherry) [28] (Figure 1B).

### 2.2. GmTRAB1 Is Induced by Multiple Abiotic Stresses

**Figure 2:**
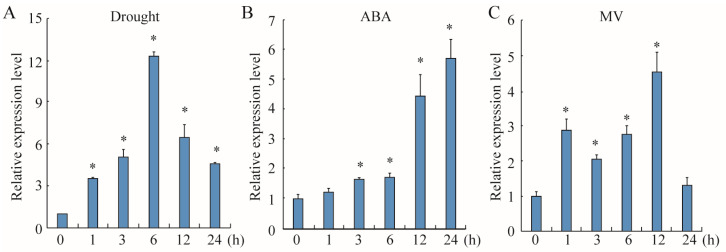
GmTRAB1 was involved in multiple stresses. The expression patterns of *GmTRAB1* under (**A**) drought, (**B**) ABA, and (**C**) MV stresses were detected by qRT-PCR. *GmTubulin* was applied as an internal control. The * suggests significant differences (* *p* < 0.05).

To assess the function of bZIPs in the modulation of soybean response to environmental challenges, we investigated the expression patterns of *GmTRAB1* under drought, ABA, and oxidative stresses using a qRT-PCR system. Drought stimulated the accumulation of the *GmTRAB1* transcript, reaching a peak at 6 h (Figure 2A). Moreover, *GmTRAB1* transcripts increased under ABA stress and reached their highest level at 24 h (Figure 2B). In addition, oxidative stress also resulted in increased *GmTRAB1* expression (Figure 2C).

### 2.3. Overexpression of GmTRAB1 Enhances Arabidopsis Resistance to Drought

**Figure 3:**
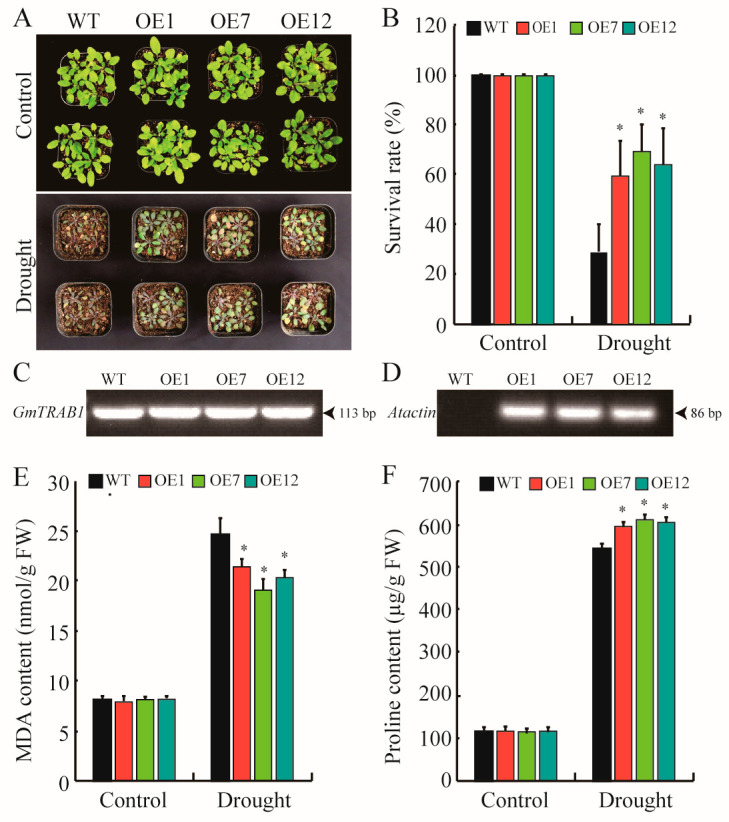
Overexpression of *GmTRAB1* leads to enhanced osmotic stress tolerance in *Arabidopsis*. (**A**) Osmotic stress tolerance assay in *GmTRAB1*-OE and WT plants. (**B**) Survival rates. (**C**,**D**) *GmTRAB1* transcripts were detected by semi-RT-qPCR. *Atactin* was applied as an internal control. (**E**) MDA content and (**F**) proline content in *GmTRAB1*-OE and control plants in response to drought. The data represent the value (±SE) of 3 independent replicates. The * suggests significant differences (* *p* < 0.05).

To characterize the role of *GmTRAB1* in plants’ resistance to drought, we constructed *GmTRAB1*-OE *Arabidopsis* using an *Agrobacterium*-mediated transformation system. The transcript levels of *GmTRAB1* in transgenic plants were detected by semi-qRT-PCR. Three independent homozygous *GmTRAB1*-OE lines (OE1, OE7, and OE12) with relatively high accumulation of *GmTRAB1* transcripts were used for the subsequent functional analysis (Figure 3C,D). *Arabidopsis* plants with similar growth characteristics were applied for osmotic stress tolerance experiments. There was no remarkable difference in plant morphology and physiological metabolism between *GmTRAB1*-OE lines and WT plants under sufficient water conditions. Severe drought stress seriously affected plant growth and physiological metabolism, and obvious differences were detected. Upon drought stress, *GmTRAB1*-OE plants displayed slighter leaf rolling and larger survival rates than the WT plants (Figure 3A,B). Drought usually causes damage to the membranes of plants, restricting their normal growth. The concentration of malonaldehyde (MDA) acted as an indicator to reflect the degree of cell membrane damage. The MDA concentration is proportional to the degree of membrane damage [1]. Proline was an osmoprotectant that functioned crucially in maintaining the stability of the cell membrane and cellular turgor pressure [29,30]. The drought-treated *GmTRAB1*-OE seedlings showed lower MDA levels and larger proline contents as compared with the WT plants (Figure 3E,F).

### 2.4. GmTRAB1 Overexpression Leads to Increased ABA Sensitivity in Transgenic Plants

**Figure 4:**
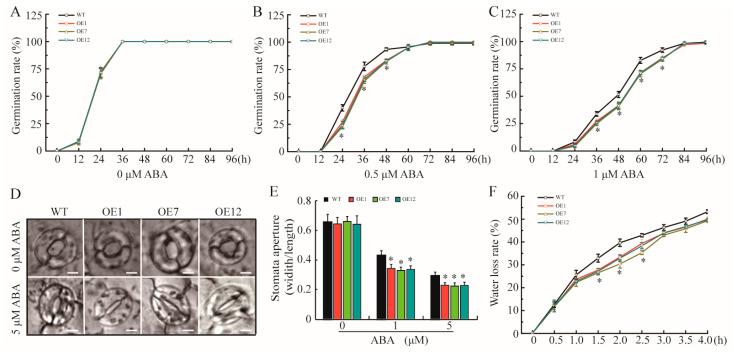
*GmTRAB1* overexpression improved ABA sensitivity in transgenic plants. (**A**–**C**) The germination rates of *GmTRAB1*-OE and WT seeds under 0, 0.5, and 1 μM ABA treatments were analyzed at 0, 12, 24, 36, 48, 60, 72, 84, and 96 h. (**D**) Phenotypes of *GmTRAB1*-OE and WT stomata upon ABA stress, Scale bars = 5 μm. (**E**) Stomatal closure of *GmTRAB1*-OE and WT upon ABA stress. The stomatal width/length ratio was utilized as the stomatal closure index. (**F**) Water loss rate. The detached leaves of *GmTRAB1*-OE and WT plants were calculated at 0, 30, 60, 90, 120, 150, 180, 210, and 240 min. The data represent the value (±SE) of 3 independent replicates. The * suggests significant differences (* *p* < 0.05).

Considering that ABA promotes the increase in *GmTRAB1* transcripts, we conducted a seed germination assay to investigate the role of *GmTRAB1* in ABA response. The *GmTRAB1*-OE and WT seeds showed similar germination rates in the absence of ABA (Figure 4A). However, exogenous ABA application inhibited seed germination, but the *GmTRAB1*-OE seeds presented significantly lower germination rates than those of the control with exposure to ABA treatments (Figure 4B,C).

ABA has been demonstrated to influence stomatal movements. In view of *GmTRAB1* overexpression improving ABA sensitivity in transgenic plants, the function of *GmTRAB1* in ABA-regulated stomatal movement was investigated. The stomatal closure index was reflected by the stomatal width/length ratio. Without ABA application, the mean of stomatal apertures between *GmTRAB1*-OE and WT plants is comparable (Figure 4D,E). Nevertheless, with the ABA application, the *GmTRAB1*-OE plants have a significantly lower mean of stomatal apertures as compared to the control plants (Figure 4D,E). Stomatal movement plays a central role in water loss and is closely related to plant adaptation to drought. Therefore, the water loss rates were also analyzed, and *GmTRAB1*-OE exhibited lower water loss rates than WT plants (Figure 4F).

### 2.5. GmTRAB1 Overexpression Confers Osmotic Stress Tolerance in Transgenic Hairy Roots of Soybean

**Figure 5:**
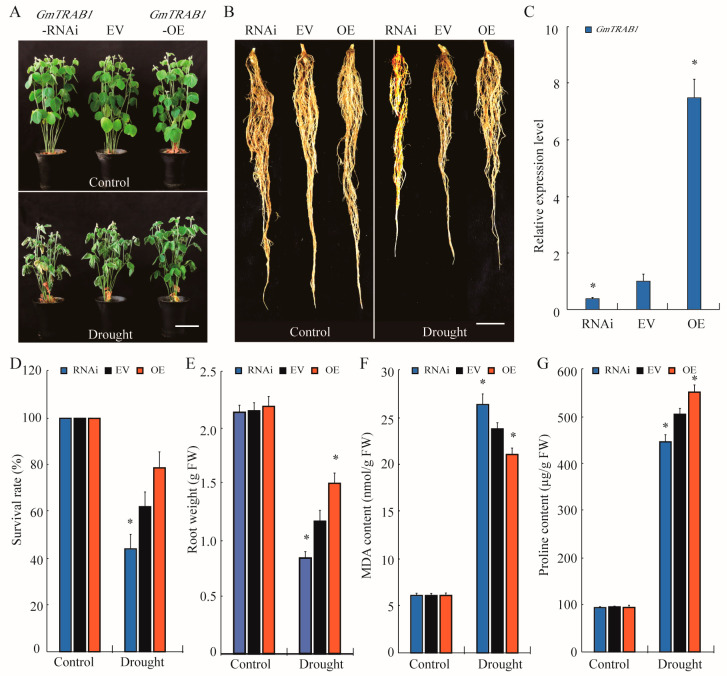
Overexpression of *GmTRAB1* results in improved osmotic stress tolerance in soybean. (**A**,**B**) Phenotypes of *GmTRAB1*-OE, *EV*, and *GmTRAB1*-RNAi soybean plants and hairy roots in response to drought. Scale bars = 3 cm. (**C**) GmTRAB1 transcripts were detected by qRT-PCR. *Gmtubulin* was applied as an internal control. (**D**) Survival rate, (**E**) fresh weight, (**F**) MDA content, and (**G**) proline content in *GmTRAB1*-OE, *EV*, and *GmTRAB1*-RNAi plants in response to drought. The data represent the value (±SE) of 3 independent replicates. The * suggests significant differences (* *p* < 0.05).

To verify the role of *GmTRAB1* in soybean resistance to drought, we generated transgenic hairy roots of soybean with *GmTRAB1*-RNA interference (RNAi) silencing or overexpressing by *Agrobacterium rhizogenes*-mediated transformation. The *GmTRAB1* expression was detected through qRT-PCR (Figure 5C). There were no significant differences in the growth and physiological characteristics of *GmTRAB1* transgenic (RNAi and OE) and empty vector (*EV*) control soybean seedlings under suitable conditions. However, drought treatment caused remarkable morphological and physiological differences among different genotypes. Upon drought stress, the *GmTRAB1*-OE seedlings presented a higher survival rate and a lighter leaf-wilting phenotype than the *EV* seedlings. In contrast, the *GmTRAB1*-RNAi plants displayed a lower survival rate and a heavier leaf-wilting phenotype (Figure 5A,D). The fresh weights of *GmTRAB1*-OE hairy roots were larger than the control roots under drought treatment, but the *GmTRAB1*-RNAi hairy roots displayed lower biomass accumulations (Figure 5B,E). Moreover, the MDA levels in *GmTRAB1*-OE hairy roots were smaller than in the control roots under drought stress, whereas the *GmTRAB1*-RNAi hairy roots presented higher MDA accumulations (Figure 5F). In addition, the *GmTRAB1*-OE hairy roots presented a higher accumulation of proline than the control roots. However, under drought stress, the *GmTRAB1*-RNAi soybean roots had lower levels of proline (Figure 5G).

### 2.6. GmTRAB1 Stimulates ROS Scavenging in Response to Drought

**Figure 6:**
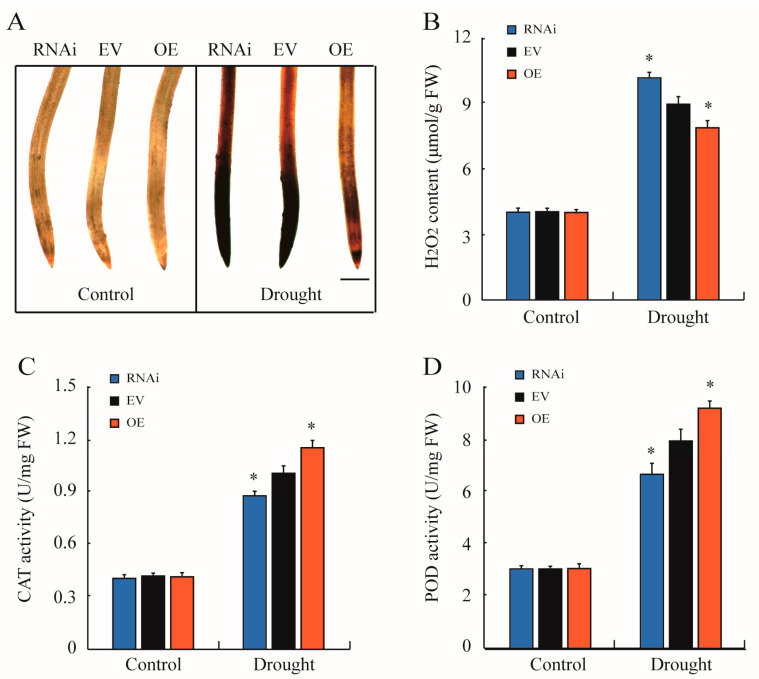
*GmTRAB1* increased ROS scavenging under drought stress. (**A**) DAB staining, scale bars = 0.1 cm; (**B**) H_2_O_2_ content; (**C**) CAT activity; and (**D**) POD activity of *GmTRAB1* transgenic (RNAi and OE) and *EV* roots under drought treatment. The data represent the value (±SE) of 3 independent replicates. The * suggests significant differences (* *p* < 0.05).

Drought caused the excessive production of reactive oxygen species (ROS) that seriously restricted the growth and development of plants. The results of DAB staining and quantitative measurement illustrated that hydrogen peroxide (H_2_O_2_) accumulation in *GmTRAB1* transgenic hairy roots was comparable to that of the *EV* control roots. With exposure to drought, the H_2_O_2_ accumulations in *GmTRAB1*-RNAi, *EV*, and *GmTRAB1*-OE hairy roots are increased. Interestingly, significant differences were observed among *GmTRAB1*-RNAi, *EV*, and *GmTRAB1*-OE hairy roots. *GmTRAB1*-RNAi seedlings accumulated a larger H_2_O_2_ content in comparison with the *EV* control roots, but the *GmTRAB1*-OE hairy roots exhibited a smaller accumulation of H_2_O_2_ (Figure 6A,B). Antioxidant enzymes play a critical role in eliminating drought-induced ROS. The activity of antioxidant enzymes was measured. After drought treatment, compared with the control, the *GmTRAB1*-OE roots displayed greater enzyme activity of catalase (CAT) and peroxidase (POD). Nevertheless, the *GmTRAB1*-RNAi roots had lower activities of CAT and POD enzymes (Figure 6C,D).

### 2.7. GmTRAB1 Increases Drought-, ABA-, and Antioxidant-Related Gene Expression in Response to Drought

**Figure 7:**
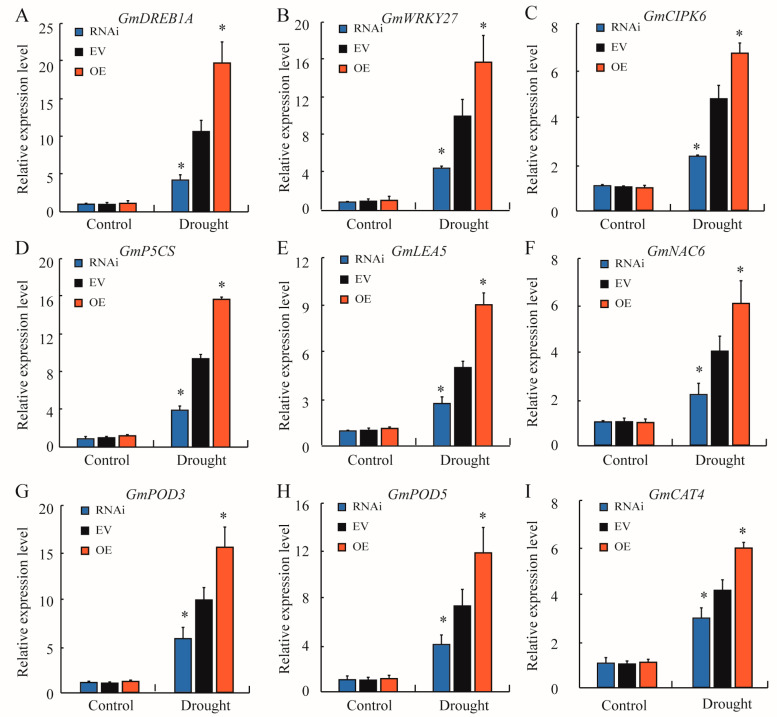
GmTRAB1 activates stress-responsive gene expression. Transcript level of (**A**) *GmDRAB1A*, (**B**) *GmWRKY27*, (**C**) *GmCIPK6*, (**D**) *GmP5CS*, (**E**) *GmLEA5*, (**F**) *GmNAC6*, (**G**) *GmPOD3*, (**H**) *GmPOD5*, and (**I**) GmCAT4 in *GmTRAB1*-RNAi, *EV*, and *GmTRAB1*-OE hairy roots under drought stress. The data represent the value (±SE) of 3 independent replicates. The * suggests significant differences (* *p* < 0.05).

To gain sight into the GmTRAB1-mediated adaptative mechanism in response to drought, the transcripts of drought-, ABA-, and antioxidant-related genes in *GmTRAB1*-RNAi, *EV*, and *GmTRAB1*-OE hairy roots upon drought stress were detected through qRT-PCR. No noticeable difference among *GmTRAB1*-RNAi, *EV*, and *GmTRAB1*-OE hairy roots was detected prior to drought treatment. Drought altered the expression of stress-related genes, and the expression of drought-induced genes (*GmDRAB1A*, *GmWRKY27*, and *GmCIPK6*), ABA-responsive genes (*GmP5CS*, *GmLEA5*, and *GmNAC6*), and antioxidant-related genes (*GmPOD3*, *GmPOD5*, and *GmCAT4*) in drought-treated hairy roots of *GmTRAB1*-OE were much greater than those of *EV* hairy roots. In contrast, lower transcript accumulation of the above genes was detected in the *GmTRAB1*-RNAi hairy roots under drought stress (Figure 7).

## 3. Discussion

Drought is usually considered the main environmental constraint that restricts crop quality and production [25]. Members of the bZIP transcription factor have been demonstrated to participate in modulating plant adaptation to drought. Nonetheless, the biological roles of bZIP proteins in enhancing soybean drought tolerance are still unclear. In this study, drought led to increased expression of the bZIP gene *GmTRAB1* (Figure 2A). Further functional analysis verified that *GmTRAB1*-OE *Arabidopsis* seedlings and soybean hairy roots displayed drought-resistant phenotypes. On the contrary, the *GmTRAB1*-RNAi soybean hairy roots were much more sensitive to drought (Figure 3 and Figure 5). These results indicated that GmTRAB1 positively regulates the osmotic stress tolerance of soybean.

The phytohormone ABA serves as a kind of pivotal signaling molecule that has critical roles in modulating plant response to drought [31]. Drought results in an increase in endogenous ABA, and the increased ABA subsequently alters the transcript accumulation of stress-related genes and stomatal movement in response to drought [32]. It has been shown that members of bZIPs are implicated in ABA-regulated adaptation to drought. For example, OsbZIP42 and OsbZIP71 have been demonstrated to modulate drought adaptation via an ABA-dependent manner [20,33]. In this assay, exogenous ABA induced the accumulation of the *GmTRAB1* transcript (Figure 2B). Moreover, with exposure to ABA treatment, the *GmTRAB1*-OE plants exhibited lower seed germination rate and smaller stomatal aperture as compared with the control plants (Figure 4B–E). Interestingly, GmTRAB1 has a role in reducing the water loss rate (Figure 4F). Additionally, upon drought stress, GmTRAB1 promoted the transcript accumulation of ABA-responsive genes, including *GmP5CS*, *GmLEA5*, and *GmNAC6* (Figure 7D–F). These results imply that GmTRAB1 is associated with modulating ABA-regulated stomatal closure and stress-responsive gene expression to regulate soybean adaptability to drought.

Drought usually stimulates the generation of ROS, including H_2_O_2_ and O_2_^−^ [34]. The overproduced ROS results in a series of cell toxicity effects, such as membrane damage, nucleic acid and protein degradation, and disruption of enzyme activity [35]. The MDA level is usually used as an important parameter to reflect the degree of cell membrane damage under stress conditions, which is negatively implicated with osmotic stress tolerance in plants [1]. The bZIP proteins have been demonstrated to influence the scavenging of ROS under adverse conditions. For example, HvbZIP21 was implicated in the scavenging of ROS to improve the osmotic stress tolerance in barley [36]. In the assay, oxidative stress induced the expression of *GmTRAB1* (Figure 2C). Subsequent quantitative measurements showed that under drought stress, compared to the control, the hairy roots of *GmTRAB1*-OE accumulated a lower content of MDA and H_2_O_2_, whereas the MDA and H_2_O_2_ levels in *GmTRAB1*-RNAi hairy roots were larger (Figure 3E, Figure 5F, and Figure 6A,B). Proline is a pivotal osmolyte that functions critically in maintaining ROS homeostasis and stabilizing cell membranes under adverse conditions [29,30]. The proline content of *GmTRAB1*-OE hairy roots under drought stress was larger. Nevertheless, the drought-treated *GmTRAB1*-RNAi hairy roots demonstrated a lower proline level. Moreover, GmTRAB1 activated the expression of *GmP5CS* (Figure 7D). Therefore, GmTRAB1 was associated with promoting the generation of proline to reduce drought-induced oxidative damage in soybean.

To mitigate oxidative damage, plants have built complicated antioxidant defense systems to remove the excessive ROS [37]. Antioxidant enzymes, including CAT, POD, and SOD, are essential parts of the antioxidant defense system [34]. The bZIPs have been reported to influence antioxidant enzyme activity to eliminate ROS under adverse conditions. For example, VvbZIP45 participated in increasing the enzyme activity of POD, SOD, and CAT to eliminate ROS under drought stress [38]. IbbZIP1 has been shown to enhance SOD activity to eliminate H_2_O_2_ under drought and salt stresses [39]. In this research, GmTRAB1 was found to increase the transcript of antioxidant enzyme genes *GmPOD3*, *GmPOD5*, and *GmCAT4* (Figure 7G–I). Furthermore, compared with the control, the enzyme activity of CAT and POD in *GmTRAB1*-OE hairy roots under drought stress was larger. Nevertheless, the *GmTRAB1*-RNAi hairy roots exhibited minor CAT activity and POD activity in response to drought (Figure 6C,D). Collectively, GmTRAB1 has a role in promoting the antioxidant defense system to eliminate ROS and alleviate drought stress in soybean.

The bZIP transcription factors have been shown to alter drought-related gene expression in response to drought. TabZIP156 was found to increase *TaDREB1A* expression in wheat in response to drought [40]. It has been demonstrated that VlbZIP30 promotes the expression of *NAC17*, *ABF2*, *PUB19*, and *PP2C9* to enhance grapevine drought tolerance [41]. In this study, GmTRAB1 functioned in improving the transcript accumulations of *GmDREB1A*, *GmCIPK6*, and *GmWRKY27* in response to drought (Figure 7A–C). DREB and WRKY transcription factors play crucial functions in increasing plant adaptability to extreme conditions [42,43]. CIPKs have been shown to participate in regulating hormone signaling, ROS scavenging, and the biosynthesis of antioxidants in response to drought [44]. The above results indicated that GmTRAB1 is implicated in stimulating the expression of drought-related genes, thereby promoting the resistance of soybean to drought.

## 4. Materials and Methods

### 4.1. Plant Materials and Growth Conditions

*Arabidopsis* Columbia-0 and soybean cultivar Williams 82 were used for plant transformation and molecular analysis. The soybean seedlings were cultured in a growth chamber at 70% relative humidity, 25 °C, and a 16 h light/8 h dark photoperiod. To analyze the expression profiles of *GmTRAB1* under drought and oxidative stress treatments, the roots of soybean seedlings (2-week-old) were immersed in 15% PEG6000 and 20 µM methyl viologen (MV) solution, respectively. The leaves of seedlings sprayed with 100 µM ABA were used to investigate *GmTRAB1* expression profiles upon ABA treatment. The leaves were collected at 0, 1, 3, 6, 12, and 24 h, respectively. *Arabidopsis* seedlings were growing in an illumination incubator at 70% relative humidity, 25 °C, and a 16 h light/8 h dark photoperiod.

### 4.2. Construction of Phylogenetic Tree

GmTRAB1 homologous proteins were retrieved from the non-redundant protein sequence database (https://blast.ncbi.nlm.nih.gov/Blast.cgi?CMD=Get&RID=JEV01RW2016, accessed on 31 October 2024). The amino acid sequence of the *Arabidopsis* bZIP proteins was downloaded from TAIR (https://www.arabidopsis.org/results?mainType=general&searchTex, accessed on 31 October 2024). The ClustalX program in MEGA7.0 software was used to perform multiple sequence alignment. The bootstrap neighbor-joining phylogenetic tree was generated by MEGA7.0 software with 500 bootstrap replicates. Ten different subgroups of AtbZIPs were used as references to classify GmTRAB1 and its homologous proteins. The accession numbers of GmTRAB1 homologous proteins and *Arabidopsis* bZIPs were listed in Table S2.

### 4.3. Quantative Real Time-PCR

The total RNA from plants was isolated with Trizol reagent, and then the genome DNA was eliminated by RNase-free DNaseI (TransGen, Beijing, China). The cDNA synthesis was performed by the TransScript One-Step RT-PCR SuperMix Kit (TransGen, Beijing, China). The qRT-PCR reactions were then conducted with a TransStart Top Green qPCR SuperMix kit using an ABI 7500 machine. The specific primers used in qRT-PCR were presented in Table S1. *Atactin* (At3g18780) and *GmTubulin* (Glyma.08G014200) were referred to as quantitative controls for *Arabidopsis* and soybean, respectively. The 2^−ΔΔCT^ method was applied for quantitative analysis. The reaction of qRT-PCR was conducted by the procedure of 95 °C (180 s), then 42 cycles of 95 °C (10 s), 57 °C (15 s), and 72 °C (45 s).

### 4.4. Subcellular Localization Analyses

To analyze the subcellular localization, we produced *GmTRAB1*-GFP constructs. *GmTRAB1*-GFP and *OsEHD4*-mCherry plasmids were introduced into the same protoplasts from *Arabidopsis*, and then the protoplasts were cultured in the dark (23 °C) for 12 h. OsEHD4-mCherry was applied as a nuclear marker [28]. The florescence signals of GFP and mCherry were detected with a confocal laser-scanning microscope.

### 4.5. Construction of Transgenic Arabidopsis and Soybean Plants

To construct *GmTRAB1*-OE *Arabidopsis* plants, the pCAMBIA1302-*GmTRAB1* plasmids were generated and then introduced into *Arabidopsis* via the *Agrobacterium*-mediated floral-dip method [31].

The transgenic soybean plants were generated using the *Agrobacterium rhizogene*-mediated soybean hairy roots transformation system, as described previously [31,42]. To generate the *GmTRAB1*-RNAi vector, a 120-bp-specific DNA fragment of *GmTRAB1* was cloned, and then these fragments were collected and connected to both sense and antisense orientations to flank the intron 6 of the rice zinc finger type family protein gene. The *GmTRAB1*-RNAi fragment was then inserted into the pCAMBIA3301 transformation vector driven by the *CaMV* 35S promoter. The full-length open reading frame of *GmTRAB1* was introduced into pCAMBIA3301 to generate the *GmTRAB1*-OE vector. The *GmTRAB1*-RNAi and *GmTRAB1*-OE vectors were transformed into K599 *Agrobacterium rhizogenes* strains, which were then applied for infecting hypocotyls of soybean to gain transgenic soybean hairy roots.

### 4.6. Osmotic Stress Tolerance Assay

For *Arabidopsis*, homozygous T3 seedlings of the *GmTRAB1*-OE and WT seedlings (1-week-old) were used for drought treatment. After normal culturing in the illumination incubator for 21 d, the *GmTRAB1*-OE and WT seedlings were subject to drought stress without irrigation for another 17 d until remarkable leaf-wilting differences were identified.

For soybean, 2-week-old *GmTRAB1*-OE, *EV*, and *GmTRAB1*-RNAi soybean seedlings were grown normally in a mixed soil (1:1 vermiculite:humus) for 7 d. These seedlings were then exposed to drought stress with 15% PEG6000 irrigation for another 10 d until remarkable differences in leaf wilting were identified.

### 4.7. Measurements of Physiological Characteristics

*Arabidopsis* seedlings (4-week-old) and soybean seedlings (3-week-old) were exposed to drought treatment with irrigating 15% PEG6000 solution for 10 d. The *Arabidopsis* leaves and soybean hairy roots were collected for measuring physiological parameters. The MDA content, proline content, hydrogen peroxide H_2_O_2_ content, peroxidase POD activity, and CAT activity were detected by their corresponding detection kits.

### 4.8. Analysis of Seed Germination, Stomatal Closure, and Water Loss Rate

For measurement of germination rate, homozygous T3 seeds of *GmTRAB1*-OE and WT were sterilized and sown on 1/2-strength Murashige and Skoog media containing 0, 0.5, and 1 µM ABA. After vernalization in the dark for 3 days, the germination rates in terms of the seed radicle emergence were counted at 0, 12, 24, 36, 48, 60, 72, 84, and 96 h, respectively.

For the water loss rate analysis, the detached rosette leaves of 4-week-old *GmTRAB1*-OE and WT Arabidopsis were measured at 0, 30, 60, 120, 180, 21, and 240 using a 1/10,000 analytical balance, respectively. The water loss rate was computed as reported previously.

For the stomatal closure analysis, the detached rosette leaves of *GmTRAB1*-OE and WT *Arabidopsis* (4-week-old) were treated with stomata opening solution (7.5 mM iminodiacetic acid, pH = 6.15, 10 mM KCl, and 10 mM MES-Tris) and exposed to bright light. Until the stomata were fully opened, the leaves were exchanged into solutions supplemented with 0, 1, and 5 µM ABA and incubated for another 2.5 h.

### 4.9. Statistical Analyses

Each experiment was independently conducted three times. Data are presented as the mean ± SE of the three independent replicates. Statistical analysis was performed with the SPSS 27.0 software. The one-way analysis of variance (ANOVA) method was applied to verify significant differences, marked as *, *p* < 0.05.

## 5. Conclusions

In this study, GmTRAB1 played a positive regulatory role in improving the osmotic stress tolerance of soybean. GmTRAB1 participated in ABA-regulated stomatal closure and stress-related gene expression to regulate the drought stress response. Furthermore, GmTRAB1 was involved in activating the antioxidant defense system to promote the scavenging of ROS under drought stress. Additionally, GmTRAB1 has a role in activating drought-related genes in drought stress response. In summary, these results will provide the theoretical basis for elucidating the molecular mechanism of bZIP transcription factors-mediated drought response in soybean.

## Data Availability

Data is contained within the article or Supplementary Material.

## Supplementary Materials

The following supporting information can be downloaded at: https://www.mdpi.com/article/10.3390/plants13213104/s1, Figure S1: Phylogenetic analysis of GmTRAB1, GsABL5, GmbZIP50, MpABL5, VuABL5, and *Arabidopsis* bZIP transcription factors. GmTRAB1 and its homologous proteins were divided into the subgroup A; Table S1: Primers used in qRT-PCR assays; Table S2: Accession numbers of bZIPs used for phylogenetic analysis.

## References

[1] Yu T.F., Liu Y., Fu J.D., Ma J., Fang Z.W., Chen J., Zheng L., Lu Z.W., Zhou Y.B., Chen M. (2021). The NF-Y-PYR module integrates the abscisic acid signal pathway to regulate plant stress tolerance. Plant Biotechnol. J..

[2] Li Z., Fu D., Wang X., Zeng R., Zhang X., Tian J., Zhang S., Yang X., Tian F., Lai J. (2022). The transcription factor bZIP68 negatively regulates cold tolerance in maize. Plant Cell.

[3] Kidokoro S., Watanabe K., Ohori T., Moriwaki T., Maruyama K., Mizoi J., Myint Phyu Sin Htwe N., Fujita Y., Sekita S., Shinozaki K. (2015). Soybean DREB1/CBF-type transcription factors function in heat and drought as well as cold stress-responsive gene expression. Plant J..

[4] Wei W., Liang D.W., Bian X.H., Shen M., Xiao J.H., Zhang W.K., Ma B., Lin Q., Lv J., Chen X. (2019). GmWRKY54 improves drought tolerance through activating genes in abscisic acid and Ca^2+^ signaling pathways in transgenic soybean. Plant J..

[5] Zhang L., Zhao L., Wang L., Liu X., Yu Z., Liu J., Wu W., Ding L., Xia C., Zhang L. (2023). TabZIP60 is involved in the regulation of ABA synthesis-mediated salt tolerance through interacting with TaCDPK30 in wheat (*Triticum aestivum* L.). Planta.

[6] Ma H., Liu C., Li Z., Ran Q., Xie G., Wang B., Fang S., Chu J., Zhang J. (2018). ZmbZIP4 contributes to stress resistance in maize by regulating ABA synthesis and root development. Plant Physiol..

[7] Jakoby M., Weisshaar B., Droge-Laser W., Vicente-Carbajosa J., Tiedemann J., Kroj T., Parcy F. (2002). bZIP transcription factors in *Arabidopsis*. Trends Plant Sci..

[8] Landschulz W.H., Johnson P.F., McKnight S.L. (1988). The leucine zipper: A hypothetical structure common to a new class of DNA binding proteins. Science.

[9] Izawa T., Foster R., Chua N.H. (1993). Plant bZIP protein DNA binding specificity. J. Mol. Biol..

[10] Nijhawan A., Jain M., Tyagi A.K., Khurana J.P. (2007). Genomic survey and gene expression analysis of the basic leucine zipper transcription factor family in rice. Plant Physiol..

[11] Pourabed E., Ghane Golmohamadi F., Soleymani Monfared P., Razavi S.M., Shobbar Z.S. (2015). Basic leucine zipper family in barley: Genome-wide characterization of members and expression analysis. Mol. Biotechnol..

[12] Li D., Fu F., Zhang H., Song F. (2015). Genome-wide systematic characterization of the bZIP transcriptional factor family in tomato (*Solanum lycopersicum* L.). BMC Genom..

[13] Zhou Y., Xu D., Jia L., Huang X., Ma G., Wang S., Zhu M., Zhang A., Guan M., Lu K. (2017). Genome-wide identification and structural analysis of bZIP transcription factor genes in *Brassica napus*. Genes.

[14] Wang J., Zhou J., Zhang B., Vanitha J., Ramachandran S., Jiang S.Y. (2011). Genome-wide expansion and expression divergence of the basic leucine zipper transcription factors in higher plants with an emphasis on Sorghum. J. Integr. Plant Biol..

[15] Wei K., Chen J., Wang Y., Chen Y., Chen S., Lin Y., Pan S., Zhong X., Xie D. (2012). Genome-wide analysis of bZIP-encoding genes in maize. DNA Res..

[16] Sun X.L., Li Y., Cai H., Bai X., Ji W., Ding X.D., Zhu Y.M. (2012). The *Arabidopsis* AtbZIP1 transcription factor is a positive regulator of plant tolerance to salt, osmotic and drought stresses. J. Plant Res..

[17] Yoshida T., Fujita Y., Sayama H., Kidokoro S., Maruyama K., Mizoi J., Shinozaki K., Yamaguchi-Shinozaki K. (2010). AREB1, AREB2, and ABF3 are master transcription factors that cooperatively regulate ABRE-dependent ABA signaling involved in drought stress tolerance and require ABA for full activation. Plant J..

[18] Xiang Y., Tang N., Du H., Ye H.Y., Xiong L.Z. (2008). Characterization of OsbZIP23 as a key player of the basic leucine zipper transcription factor family for conferring abscisic acid sensitivity and salinity and drought tolerance in rice. Plant Physiol..

[19] Lu G., Gao C., Zheng X., Han B. (2009). Identification of OsbZIP72 as a positive regulator of ABA response and drought tolerance in rice. Planta.

[20] Joo J., Lee Y.H., Song S.I. (2019). OsbZIP42 is a positive regulator of ABA signaling and confers drought tolerance to rice. Planta.

[21] Bi C., Yu Y., Dong C., Yang Y., Zhai Y., Du F., Xia C., Ni Z., Kong X., Zhang L. (2021). The bZIP transcription factor TabZIP15 improves salt stress tolerance in wheat. Plant Biotechnol. J..

[22] Yao L., Hao X.Y., Cao H.L., Ding C.Q., Yang Y.J., Wang L., Wang X.C. (2020). ABA-dependent bZIP transcription factor, CsbZIP18, from *Camellia sinensis* negatively regulates freezing tolerance in *Arabidopsis*. Plant Cell Rep..

[23] Gai W.X., Ma X., Qiao Y.M., Shi B.H., Ul Haq S., Li Q.H., Wei A.M., Liu K.K., Gong Z.H. (2020). Characterization of the bZIP transcription factor family in pepper (*Capsicum annuum* L.): CabZIP25 positively modulates the salt tolerance. Front. Plant Sci..

[24] Wei W., Lu L., Bian X.H., Li Q.T., Han J.Q., Tao J.J., Yin C.C., Lai Y.C., Li W., Bi Y.D. (2023). Zinc-finger protein GmZF351 improves both salt and drought stress tolerance in soybean. J. Integr. Plant Biol..

[25] Yuan X.B., Jiang X.Y., Zhang M.Z., Wang L.F., Jiao W., Chen H.T., Mao J.R., Ye W.X., Song Q.X. (2024). Integrative omics analysis elucidates the genetic basis underlying seed weight and oil content in soybean. Plant Cell.

[26] Zhang Z., Ma J., Yang X., Liu Z., Liu Y., Liu X., Liang S., Duan Z., Wang Z., Yang X. (2024). Natural allelic diversities of GmPrx16 confer drought tolerance in soybean. Plant Biotechnol. J..

[27] Zhang M., Liu Y., Shi H., Guo M., Chai M., He Q., Yan M., Cao D., Zhao L., Cai H. (2018). Evolutionary and expression analyses of soybean basic Leucine zipper transcription factor family. BMC Genom..

[28] Gao H., Zheng X.M., Fei G., Chen J., Jin M., Ren Y., Wu W., Zhou K., Sheng P., Zhou F. (2013). Ehd4 encodes a novel and oryza-genus-specific regulator of photoperiodic flowering in rice. PLoS Genet..

[29] Gou C., Huang Q., Rady M.M., Wang L., Ihtisham M., El-Awady H.H., Seif M., Alazizi E.M.Y., Eid R.S.M., Yan K. (2023). Integrative application of silicon and/or proline improves Sweet corn (*Zea mays* L. saccharata) production and antioxidant defense system under salt stress condition. Sci. Rep..

[30] Hayat S., Hayat Q., Alyemeni M.N., Wani A.S., Pichtel J., Ahmad A. (2012). Role of proline under changing environments: A review. Plant Signal. Behav..

[31] Xu M., Li H., Liu Z.N., Wang X.H., Xu P., Dai S.J., Cao X., Cui X.Y. (2021). The soybean CBL-interacting protein kinase, GmCIPK2, positively regulates drought tolerance and ABA signaling. Plant Physiol. Biochem..

[32] Liu Y., Yu T.F., Li Y.T., Zheng L., Lu Z.W., Zhou Y.B., Chen J., Chen M., Zhang J.P., Sun G.Z. (2022). Mitogen-activated protein kinase TaMPK3 suppresses ABA response by destabilising TaPYL4 receptor in wheat. New Phytol..

[33] Liu C., Mao B., Ou S., Wang W., Liu L., Wu Y., Chu C., Wang X. (2014). OsbZIP71, a bZIP transcription factor, confers salinity and drought tolerance in rice. Plant Mol. Biol..

[34] Xiang Y., Bian X., Wei T., Yan J., Sun X., Han T., Dong B., Zhang G., Li J., Zhang A. (2021). ZmMPK5 phosphorylates ZmNAC49 to enhance oxidative stress tolerance in maize. New Phytol..

[35] Qi J., Song C.P., Wang B., Zhou J., Kangasjärvi J., Zhu J.K., Gong Z. (2018). Reactive oxygen species signaling and stomatal movement in plant responses to drought stress and pathogen attack. J. Integr. Plant Biol..

[36] Pan R., Buitrago S., Feng Z., Abou-Elwafa S.F., Xu L., Li C., Zhang W. (2022). HvbZIP21, a novel transcription factor from wild barley confers drought tolerance by modulating ROS scavenging. Front. Plant Sci..

[37] Cui X.Y., Gao Y., Guo J., Yu T.F., Zheng W.J., Liu Y.W., Chen J., Xu Z.S., Ma Y.Z. (2019). BES/BZR transcription factor TaBZR2 positively regulates drought responses by activation of TaGST. Plant Physiol..

[38] Niu S., Gu X., Zhang Q., Tian X., Chen Z., Liu J., Wei X., Yan C., Liu Z., Wang X. (2023). Grapevine bZIP transcription factor bZIP45 regulates VvANN1 and confers drought tolerance in *Arabidopsis*. Front. Plant Sci..

[39] Kang C., Zhai H., He S., Zhao N., Liu Q. (2019). A novel sweetpotato bZIP transcription factor gene, IbbZIP1, is involved in salt and drought tolerance in transgenic *Arabidopsis*. Plant Cell Rep..

[40] Bu Y., Yu Y., Song T., Zhang D., Shi C., Zhang S., Zhang W., Chen D., Xiang J., Zhang X. (2024). The transcription factor TabZIP156 acts as a positive regulator in response to drought tolerance in *Arabidopsis* and wheat *(Triticum aestivum* L.). Plant Physiol. Biochem..

[41] Tu M., Wang X., Zhu Y., Wang D., Zhang X., Cui Y., Li Y., Gao M., Li Z., Wang Y. (2018). VlbZIP30 of grapevine functions in dehydration tolerance via the abscisic acid core signaling pathway. Hortic. Res..

[42] Wang F., Chen H.W., Li Q.T., Wei W., Li W., Zhang W.K., Ma B., Bi Y.D., Lai Y.C., Liu X.L. (2015). GmWRKY27 interacts with GmMYB174 to reduce expression of GmNAC29 for stress tolerance in soybean plants. Plant J..

[43] Zhou Y., Chen M., Guo J., Wang Y., Min D., Jiang Q., Ji H., Huang C., Wei W., Xu H. (2020). Overexpression of soybean *DREB1* enhances drought stress tolerance of transgenic wheat in the field. J. Exp. Bot..

[44] Deng J., Yang X., Sun W., Miao Y., He L., Zhang X. (2020). The calcium sensor CBL2 and its interacting kinase CIPK6 are involved in plant sugar homeostasis via interacting with tonoplast sugar transporter TST2. Plant Physiol..

