# Supplementary material for: GmTRAB1, a Basic Leucine Zipper Transcription Factor, Positively Regulates Drought Tolerance in Soybean (Glycine max. L)
Source: Plants (Basel). 2024 Nov 4;13(21):3104. doi: 10.3390/plants13213104 (PMC11548361; doi:10.3390/plants13213104)
Supplement: Supplementary file 1 [file plants-13-03104-s001.zip › plants-3275826-supplementary.pdf]

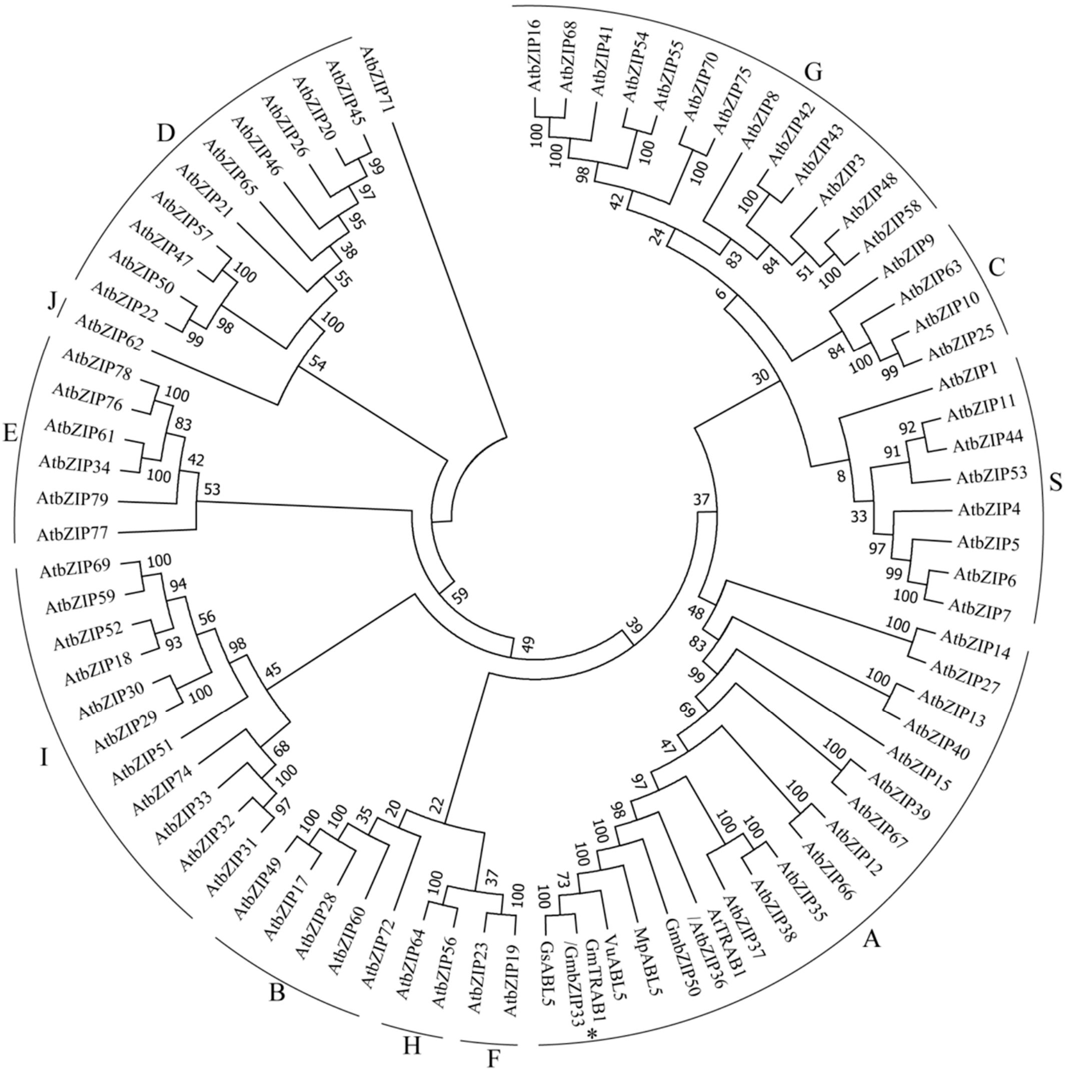

Figure S1 Phylogenetic analysis of GmTRAB1, GsABL5, GmbZIP50, MpABL5, VuABL5, and *Arabidopsis* bZIP transcription factors. GmTRAB1 and its homologous proteins were divided into the subgroup A.

Table S1 Primers used in qRT-PCR assays

Gene		Primers
Atactin	Forward	5'- GAAATCACAGCACTTGCACC -3'
At3g18780	Reverse	5'-TGGAATGTGCTGAGGGAAGC-3'
Gmtubulin	Forward	5'-GAGGCAAGATGAGCACCAAG-3'
Glyma.08G014200	Reverse	5'-ACGGAACATTTCCTGAATGGAG-3'
GmTRAB1	Forward	5'- GTTGGAGGAGTTTTTGGTCAGA -3'
Glyma.04G039300	Reverse	5'- CAACGCGAGACAGATCTACGA-3'
GmDREB1B	Forward	5'- AGTGAGGCAGAGGAACGGGA-3'
Glyma.10G067000	Reverse	5'- AATGTTCCGAGCCACACCC-3'
GmWRKY27	Forward	5'-GTAACAACAGGTTCCAACCGTTCA-3'
Glyma.15G003300	Reverse	5'-CTTCTGGTGATTCAGTTTTGGGATT-3'
GmCIPK6	Forward	5'-AAACCGCCAGACTTTACTTCC -3'
Glyma.09G098000	Reverse	5'-GCCTGAGATGCTCGGAGAAAG-3'
GmP5CS	Forward	5'- GCGGATCCTTCTCGGAGTTT -3'
Glyma.18G034300	Reverse	5'- TCAATTTGCGGTAGCGGAGT -3'
GmLEA5	Forward	5'-CCGATGTATCGGTAAGAGT-3'
Glyma.17G027400	Reverse	5'-AGGCTTTTGAACCATCTC -3'
GmNAC6	Forward	5'-TGGGATGATGATGTTGGACTCTTAT-3'
Glyma.06G195500	Reverse	5'-GGTGTTGCTGTTGATGCTGATAT-3'
GmPOD3	Forward	5'-GCATTTCCATGATTGTTTTGTAAG-3'
Glyma.13G306900	Reverse	5'-CAGCCTGATTGGTTGTTGAGTTC-3'
GmPOD5	Forward	5'-AGTGTTTCATCATTGGCTTCAGC-3'
Glyma.10G191700	Reverse	5'-ATTCTTATGAGACCCGCAGCTATG-3'
GmCAT4	Forward	5'-TCAATTCTCCCTTCTGGACTACA-3'
Glyma.04G017500	Reverse	5'-AAAGAAACCCTTTGCACTAGCG-3'

Table S2. Accession numbers of bZIPs used for phylogenetic analysis

Gene name	Gene ID	Protein name	Protein ID
AtbZIP1	At5g49450	GsABL5	XP_028227653.1
AtbZIP10	At4g02640	MpABL5	RDX68365.1
AtbZIP11	At4g34590	VuABL5	XP_027903191.1
AtbZIP12	At2g41070	GmbZIP50	NP_001341123.1
AtbZIP13	At5g44080		
AtbZIP14	At4g35900		
AtbZIP15	At5g42910		
AtbZIP16	At2g35530		
AtbZIP17	At2g40950		
AtbZIP18	At2g40620		

AtbZIP19	At4g35040		
AtbZIP2	At2g18160		
AtbZIP20	At5g06950		
AtbZIP21	At1g08320		
AtbZIP22	At1g22070		
AtbZIP23	At2g16770		
AtbZIP24	At3g51960		
AtbZIP25	At3g54620		
AtbZIP26	At5g06960		
AtbZIP27	At2g17770		
AtbZIP28	At3g10800		
AtbZIP29	At4g38900		
AtbZIP3	At5g15830		
AtbZIP30	At2g21230		
AtbZIP31	At2g13150		
AtbZIP33	At2g12900		
AtbZIP34	At2g42380		
AtbZIP35	At1g49720		
AtbZIP36	At1g45249		
AtbZIP37	At4g34000		
AtbZIP38	At3g19290		
AtbZIP39	At2g36270		
AtbZIP4	At1g59530		
AtbZIP40	At1g03970		
AtbZIP41	At4g36730		
AtbZIP42	At3g30530		
AtbZIP43	At5g38800		
AtbZIP44	At1g75390		
AtbZIP45	At3g12250		
AtbZIP46	At1g68640		
AtbZIP47	At5g65210		
AtbZIP48	At2g04038		
AtbZIP49	At3g56660		
AtbZIP5	At3g49760		
AtbZIP50	At1g77920		
AtbZIP51	At1g43700		
AtbZIP52	At1g06850		
AtbZIP53	At3g62420		
AtbZIP54	At4g01120		
AtbZIP55	At2g46270		
AtbZIP56	At5g11260		
AtbZIP57	At5g10030		
AtbZIP58	At1g13600		

AtbZIP59	At2g31370		
AtbZIP6	At2g22850		
AtbZIP60	At1g42990		
AtbZIP61	At3g58120		
AtbZIP62	At1g19490		
AtbZIP63	At5g28770		
AtbZIP64	At3g17609		
AtbZIP65	At5g06839		
AtbZIP66	At3g56850		
AtbZIP67	At3g44460		
AtbZIP68	At1g32150		
AtbZIP69	At1g06070		
AtbZIP7	At4g37730		
AtbZIP70	At5g60830		
AtbZIP71	At2g24340		
AtbZIP72	At5g07160		
AtbZIP74	At2g21235		
AtbZIP75	At5g08141		
AtbZIP8	At1g68880		
AtbZIP9	At5g24800		
